# Neural Correlates of Psychotherapy in Anxiety and Depression: A Meta-Analysis

**DOI:** 10.1371/journal.pone.0074657

**Published:** 2013-09-11

**Authors:** Irene Messina, Marco Sambin, Arianna Palmieri, Roberto Viviani

**Affiliations:** 1 Department of Philosophy, Sociology, Education and Applied Psychology, University of Padua, Padua, Italy; 2 Department of Psychiatry and Psychotherapy III, University of Ulm, Ulm, Germany; 3 Institute of Psychology, University of Innsbruck, Innsbruck, Austria; King's College London, United Kingdom

## Abstract

Several studies have used neuroimaging methods to identify neural change in brain networks associated to emotion regulation after psychotherapy of depression and anxiety. In the present work we adopted a meta-analytic technique specific to neuroimaging data to evaluate the consistence of empirical findings and assess models of therapy that have been proposed in the literature. Meta-analyses were conducted with the Activation Likelihood Estimation technique, which evaluates the overlap between foci of activation across studies. The analysis included 16 studies found in Pubmed (200 foci of activation and 193 patients). Separate meta-analyses were conducted on studies of 1) depression, post-traumatic stress disorder and panic disorder investigated with rest state metabolism (6 studies, 70 patients); 2) depression, post-traumatic stress disorder and panic disorder investigated with task-related activation studies (5 studies, 65 patients); 3) the previous studies considered jointly; and 4) phobias investigated with studies on exposure-related activation (5 studies, 57 patients). Studies on anxiety and depression gave partially consistent results for changes in the dorsomedial prefrontal cortex and in the posterior cingulated gyrus/precuneus. Several areas of change in the temporal lobes were also observed. Studies on the therapy of phobia were consistent with a reduction of activity in medial temporal areas. The cluster of change in the prefrontal cortex may refer to increased recruitment of control processes, as hypothesized by influential models of emotion regulation changes due to psychotherapy. However, not all areas associated with controlled emotion regulation were detected in the meta-analysis, while involvement of midline structures suggested changes in self-related information processing. Changes in phobia were consistent with reduced reactivity to phobic stimuli.

## Introduction

In the last few years, neuroimaging approaches to psychotherapy research have provided considerable evidence of changes in brain networks accompanying treatment [1]. The aim of neuroimaging studies is to characterize the operation of different psychotherapy approaches in terms of their neural substrates by tracing the impact of treatment on networks involved in emotional processing [2], [3]. Because of the relative lack of specificity of symptomatic improvement, the importance of neuroimaging techniques may consist in a more specific characterization of the mechanisms of therapy than the one obtainable by observing therapy outcome.

Several reviews of neuroimaging studies on psychotherapy of depression and anxiety have noted that treatment results in changes in brain areas that play important roles in emerging models of emotional processing and emotion regulation [4]–[6]. Emotion regulation is thought to involve interactions between components of a limbic-cortical system that mediates the capacity to control emotion [7], [8]. In particular, neuroimaging studies on emotion regulation have reported activation of the prefrontal cortex, which is also active in hallmark studies of executive control [9], in association with recruitment of emotion control processes [10]. Further studies confirmed that the same prefrontal areas are active during these attempts at control and while executing demanding cognitive tasks [11], [12]. Based on these findings, a model of emotion regulation has been proposed in which the prefrontal cortex has an inhibitory effect on areas associated with emotional reactivity, such as the amygdala, resulting in decreased emotional experience in participants [13], [14]. This is an example of dual-process models, widely employed in psychology and neuroscience, that postulates the existence of top-down, voluntary, controlled processes and bottom-up, non-voluntary, automatic processes [15].

This model is supported by numerous important findings. Several studies have independently reported increased activation in limbic areas, such as the amygdala, during the exposure to emotionally arousing stimuli in disorders characterized by affective dysregulation and impulsivity [16]–[20]. Other studies have shown that activity in these circuits may be modulated by cognitive function [21], [21]–[23] and by voluntary attempts to control one's affective reaction to emotional stimuli [24]–[31]. Furthermore, remission appears to be characterized by recovery of the balance between activity in prefrontal and limbic areas [20].

Taken together, these findings support the validity of a core construct of emotional dysregulation generally applicable to affective disorders and their therapy, and of the role of cognitive control processes in containing enhanced reactivity to emotional stimulation. Applying a model based on this construct to summarize a wide range of findings, DeRubeis and colleagues [32] have argued that the neural correlate of psychotherapy of depression may consist in increased activation of prefrontal areas when participants are exposed to emotional stimuli, representing successful recruitment of control processes in the context of emotion regulation. An analogous model has been proposed for anxiety disorders and their therapy [1]. However, others have noted the existence of experimental findings that are not always consistent with the hypothesis of increased prefrontal activation accompanying improvement. For example, reports of both increases and decreases in prefrontal regions are present in the literature [2], [33]. Moreover, the hypothesis of prefrontal activity increases as a marker of effective emotion regulation is inconsistent with studies of depressed patients that found prefrontal hyper-activity in association with dysfunctional thoughts such as rumination [34]. Similarly, in studies of treatment of post-traumatic stress disorder, decreased prefrontal activity has been associated with less intrusive traumatic memories [35].

To address the issue of direction of change in studies of psychotherapies, it is important to consider the existence of two large groups of experimental settings: studies of changes in baseline activity, largely based on quantitative approaches such as PET, and studies of changes of task-related activation, based on more recent BOLD-EPI methodologies. Studies of task-related activation test the model of emotion regulation directly, either by eliciting signal in limbic structures associated with the exposure to emotionally arousing stimuli (to assess emotional reactivity), or by investigating neural correlates of cognitive demanding tasks where distracters are made more disruptive by manipulating their emotional content (to assess efficiency of cognitive regulation).

In contrast, studies of baseline metabolism or resting state may be indicative of mental activity free of the constraints of tasks or stimuli (‘default mode’ function). This activity is believed to arise at least in part from self-referential and unconstrained thinking, such as thoughts arising spontaneously while resting in the scanner [36]–[40]. Areas that are more active during the default mode function are typically deactivated by focussed thinking tasks. It therefore seems possible that spontaneous and instructed thought reveal complementary aspects of mental activity. Importantly, the antagonistic relationship between task-related activity and default mode functioning has been shown in several studies to be affected by disorders of affect [41]–[44]. The pertinence of these findings rests on the possible importance of controlling spontaneous thinking in the default mode in disorders of affect. Therefore, even if often conceived and interpreted without considering the possible functional characterization of the resting state, the studies on rest activity may be assessed in view of possible areas of concordance or contrast with task-related studies. Furthermore, the structures more often involved in resting state studies of depression or anxiety are areas in the prefrontal and limbic regions of the brain that appear to be relevant to the model of emotion regulation [45].

The present work complements the several reviews that have discussed the findings of neuroimaging studies on psychotherapy [1], [2], [4]–[6], [46] by adopting a more formal approach based on meta-analytic techniques to summarize and evaluate the consistence of empirical findings and their relation to theoretical claims on the action of therapy [47], [48]. In neuroimaging studies of the psychotherapy of affective disorders, the use and refinement of formal meta-analytic technique may play an important role. Despite the individual importance of these studies, there are several reasons why a neuroimaging study of psychotherapy may not be conclusive when considered in isolation. The cost of scans and therapies, the problems ensuing from recruiting adequately sized samples and the losses at follow-up, the modest effects associated with second or third-order interactions typically representing the contrast of interest in such studies, make it difficult to demonstrate the existence of strong effects when applying corrections for multiple tests for the whole brain. Meta-analyses, which are designed to detect consistent effects across several experiments, may lead to progress in the face of these methodological difficulties.

In the present study we adopted Activation Likelihood Estimation [ALE], a meta-analytic technique specifically developed for neuroimaging studies [49], to summarize the results of longitudinal studies about psychotherapy with the aim of clarifying the neural mechanisms of psychotherapy change for both baseline and task-related studies. We included task-related studies that investigated the effects associated with psychotherapy in balancing controlled processes of emotion regulation and automatic processes of emotional reactivity in emotional disorders. Both depression and anxiety disorders were considered in the present analysis in view of the existence of overlapping symptoms and diagnostic criteria [5], the high co-morbidity [50], and because the same core construct is adopted in the literature to model the circuits involved in these disorders and their therapy. Both disorders are thought to involve similar changes in processes localized to the prefrontal cortex, the amygdala, and the hippocampus, and a loss of balance between emotion regulation and reactivity [51], in accord to the previously mentioned dual-process model of emotion regulation. Moreover, both disorders respond to similar treatments, which include not only cognitive-behavioural therapy, but also antidepressant targeting the serotonergic system [5].

Within the group of task-related activation studies, we conducted a separate meta-analysis of studies of the psychotherapy of specific phobias. This choice was motivated by specific characteristics of these therapeutic approaches and of the studies in the literature, suggesting possible systematic differences relative to studies of depression and other forms of anxiety, even if the core construct of affect dysregulation may be commonly applicable. Therapies of specific phobias are characterized by exposure to the same stimulus that is used in the neuroimaging studies to activate the brain-behavioural phenotype of this disorder (for example, spiders are shown in the scanner to aracnophobic patients). This contrasts with paradigms used in other studies, which cannot make recourse to stimuli of this degree of specificity (i.e., no therapy of depression involves desensitization to faces showing emotional expressions, which are commonly used in the scanner to probe reactivity to emotionally arousing stimuli). Accordingly, studies of the effects of the psychotherapy of phobias can focus more narrowly on the neural substrate activated by the stimulus directly addressed by therapy. This differs from common models of the effects of psychotherapy, which posit effects on prefrontal areas onto which control processes are mapped [1], [32], [51].

Referring to the dual-process model of dysregulation in affective disorders and their therapy, we were specifically interested in addressing the following issues:

1. to test the prediction of dual-process models of emotion regulation, according to which psychotherapy is associated with increases in activity in these prefrontal areas, accompanied by a decrement of activity in the limbic system, in task-related studies.
2. the involvement of specific areas in the prefrontal cortex and the limbic system in accounting for changes ensuing during therapy in resting state studies;
3. to verify the possible concordance of areas detected in task-related and baseline types of studies.

Note that the direction of changes cannot be the same in task-related and resting state studies. First, in a task-related activation study, the control condition enters on the subtraction side in the contrast task vs. control. To the extent that this control condition is more akin to a baseline [52], changes attributable to it will appear with reversed sign in the contrast effect. This reasoning is confirmed by consistent empirical evidence showing that decrements in baseline levels result in larger task-related activations, and vice-versa [53]–[55]. Second, areas associated with semantic and emotional processing, mostly located in the ventral part of the brain and related to the default network system on the one hand, and areas typically activated by tasks are functionally characterized by antagonistic activation patterns [14], [56]. Therefore, areas in the prefrontal cortex appear to react with changes of different sign in the presence of a task, depending on the relationship of the area in question with the default network system. This antagonistic relationship is consistent with the different mode of function required by thought directed by the execution of a task on the one hand and self-referential or spontaneous thought on the other [56]. For this reason, it is not enough to note the existence in the literature of changes in different directions detected in prefrontal areas to conclude against the consistency of changes associated with psychotherapy.

To address this issue, we computed a first group of meta-analyses in which baseline and task-related studies were examined separately. This first series of analyses allows examining the data without reference to the model of the possible antagonistic relationship between instructed and spontaneous thought. A final meta-analysis including both studies was also carried out to quantify evidence of concordance between these two types of studies, after changing the sign of effects of baseline studies.

The evidence on the existence of a form of affective dysregulation shared between depression and anxiety, discussed above, does not imply the absence of psychopathological criteria that may differentiate between these disorders (or indeed between subtypes within them). However, there are very few studies in the neuroimaging literature of psychotherapy addressing aspects of psychopathology that do not fall within the scope of the emotion dysregulation construct (a rare example of such studies is [57], which addresses the symptom of anhedonia). We excluded these few studies from the present work, both because their small number prevents the effective application of meta-analytic techniques, and because it seems to us that the emotion dysregulation construct is of such importance for the clinical neuroscience of affect to warrant specific analysis. The same reasoning led us to neglect the systematic analysis of studies categorized by disorder, as at present there are not enough studies in each nosological group to yield conclusive results.

## Materials and Methods

### Literature Search and Studies Selection

Neuroimaging studies comparing patterns of activity in psychiatric patient before and after psychotherapy were collected through searches in PUBMED (http://www.ncbi.nlm.nih.gov/pubmed/) using the keywords “psychotherapy neuroimaging”. The literature showed considerable heterogeneities across studies in experimental design and therapy approach. To limit the meta-analysis to studies of sufficient consistency of scope, a selection was carried out following criteria base on (a) the diagnostic category, (b) the psychotherapy approaches, (c) the experimental design and (d) methodological issues.

- Depression and anxiety disorders were included. Even if obsessive-compulsive disorder is classified as an anxiety disorder by current diagnostic systems [58], it was excluded from the meta-analysis because of its specific neural correlates, which presumably set it apart from other disorders in the group [59].
- We included studies investigating neural correlates of psychotherapy regardless of the specific therapy approach. This followed our interest in verifying the common model of emotion regulation adopted by the authors of these studies. Furthermore, empirical evidence in the psychotherapy research field suggests the existence of nonspecific therapeutic factors, common to all psychotherapy approaches, whose relevance to outcome may be higher than or as high as approach-specific therapeutic factors [60], [61].
- Task-related studies were included that presented emotionally arousing stimuli as targets or distractors to a demanding cognitive task, as both fall within the framework of the emotion regulation model. One study did not satisfy this criterion, due to its investigation of a reward paradigm aimed at assessing changes in depressive anhedonia [57].
- With respect of the method followed in the study, criteria for inclusion were: longitudinal experimental design, general linear model in neuroimaging data analysis, reported coordinates of activation foci in 3D coordinates (x, y, z) in stereotactic space and whole brain statistical analysis. One study on depression [62] and two studies on specific phobias [63], [64] were excluded because they did not report brain coordinates of activation foci. One study on depression was excluded because of its limitation to ROI analyses without additional whole brain analysis [65].

The studies identified using the criteria described above were 16 (listed in Table 1), yielding a total of 200 foci of activation and 193 patients.

**Table 1:** Studies included in the meta-analysis.

Studies		Patients	Design	Therapy	N sessions	Hypotheses	Task	Stimuli	Contrast	WC	HC		PC	N Foci
***Resting State studies on depression and anxiety***														
1	Brody et al., 2001 [92]	Depression (N = 14)	PET	IPT	12	Increase of DLPFC and temporal lobeactivation and decrease of VLPFC andlimbic system activation	–	–	Pre vs. Post	–		16	10	7
2	Goldapple et al., 2004 [34]	Depression (N = 14)	PET	CBT	15–20	Modulation of cortical top-down vs.sub-cortical bottom-up mechanisms.	–	–	Pre vs. Post	–		–	13	16
3	Prasko et al., 2004 [93]	Panic (N = 6)	PET	CBT	18	Changes in PFC, temporal lobes, insula,amygdala and hippocampus.	–	–	Pre vs. Post	–		–	6	29
4	Sakai et al., 2006 [94]	Panic (N = 11)	PET	CBT	10	Modulation of cortical top-down vs. sub-cortical bottom-up mechanisms of thepanic neurocircuitry, as effect ofdecrease in cognitive misattributionand emotional reactions.	–	–	Pre vs. Post	–		–	–	8
5	Kennedy et al., 2007 [95]	Depression (N = 17)	PET	CBT	8–16	Modulation of prefrontal areas associatedto altered cognition and connectedwith the limbic system.	–	–	Pre vs. Post	–		–	14	18
6	Lindauer et al., 2008 [35]	PTSD (N = 10)	SPECT	BEP	16	Change of blood flow in the prefrontalcortex as effects of fear responsessuppression increase, through the relationshipbetween MPFC and amygdala	–	–	Group × Time	10		15	–	1
***Emotional-cognitive tasks studies on depression and anxiety***														
7	Felmingham et al., 2007 [96]	PTSD (N = 8)	fMRI	CBT	8	Increase of VLPFC control over amygdala- based fear processing	Response toemotional stimuli	Fear facialexpression vs.neutral	Pre vs. Post (trials with fear stimuli)	–		–	–	7
8	Fu et al., 2008 [97]	Depression (N = 16)	fMRI	CBT	16	Decrease of amygdala activation inresponse to implicit negativefacial expression.	Gender detection	Low, medium,high sad facialexpression	Group × Time	–		16	–	48
9	Beutel et al., 2010 [98]	Panic (N = 12)	fMRI	PDT	4	Normalization of the reciprocal suppression of LPFC and emotion-related limbic areas.	Go/No-go	Anxiety anddepressionrelated words vs.neutral words	Group × Time (trials with emotional stimuli)	–		18	–	2
10	Dichter et al., 2010 [99]	Depression (N = 12)	fMRI	BAT	11	Normalization of prefrontal and limbicdysfunction associated to deficits incognitive control and hyper-responsibility to sad events.	Target/no-target task	Sad pictures	Group × Time (In response to sad vs. neutral pictures)	–		15	–	23
11	Buchheim et al., 2012 [100]	Depression (N = 16)	fMRI	PDT	90–210	Normalization of emotional reactivity(amygdala activation decrease) andemotion regulation (changes inprefrontal areas)	Response toemotional stimuli	Attachment-related pictures	Group × Time	–		17	–	3
***Symptoms provocation studies on specific phobia***														
12	Furmark et al., 2002 [101]	Social phobia (N = 6)	PET	ET	8	Habituation in amygdala andhippocampus reactivity tosocial situation.	–	Speech task	Group × Time	–		–	6	6
13	Paquette et al., 2003 [102]	Spider phobia (N = 12)	fMRI	ET	4	Normalization of prefrontal cortex,hippocampus and visualcortex activity.	Phobic vs.Neutral videos	Observation	Pre vs. Post	–		13	–	10
14	Straube et al., 2006 [103]	Spider phobia (N = 13)	fMRI	ET	2	Attenuation of hyper-activity in theParahippocampal gyrus and DLPFC	Phobic vs. Neutral videos	Observation	Group × Stimuli	12		14	–	7
15	Schienle et all., 2007 [104]	Spider phobia (N = 14)	fMRI	ET	1	Normalization of prefrontal cortex,hippocampus and visual cortex activity due to deconditioningand cognitive misattribution reductions	Phobic vs. Control Stimuli	Observation	Pre vs. Post	12		25	–	3
16	Hauner et al., 2012 [105]	Spider phobia (N = 12)	fMRI	ET	1	Normalization of top-downinfluences whereby cognitive control is exertedover fear responses viaPFC-mediated inhibition of the amygdala.	Phobic vs. Neutral pictures	Observation	Pre vs. Post	6		–	–	12

PET = Positron Emission Tomography; fMRI = Funcional Magnetic Resonance Imaging; SPECT = Single-Photon Emission Computed Tomography.

WC = Waiting list Controls; HC = Healthy Controls; PC = Pharmacotherapy Controls.

CBT = Cognitive-Behavioural Therapy, PDT = Psychodynamic Therapy, IPT = Interpersonal Therapy, BEP = Brief Eclectic Psychotherapy; BAT = Behavioural Activation Therapy, ET = Exposure Therapy.

PTSD = Post-Traumatic Stress Disorder.

The following meta-analyses were conducted:

- a meta-analysis to evaluate the contrast pre- versus post-psychotherapy limited to resting state studies;
- a meta-analysis to evaluate the contrast pre- versus post-psychotherapy limited to studies on emotional-cognitive tasks;
- a meta-analysis to evaluate the contrast pre- versus post-psychotherapy considering resting state studies and studies on emotional-cognitive tasks considered conjunctly;
- an explorative meta-analysis to evaluate the contrast pre- versus post-psychotherapy limited to exposure therapies studies on phobias.

Effects for all contrasts were evaluated in both directions (increased and decreased effects after therapy).

### Activation Likelihood Estimation

Activation Likelihood Estimation (ALE) evaluates the overlap between foci of activation found in different studies and treats them as probability distributions centred at the coordinates reported [66]. In this approach, each reported peak is converted into a Gaussian kernel centered at the peak coordinates, which is combined with the kernels generated by the other peaks to generate an estimate of peak density across the volume. A threshold for statistical significance of this density estimate is established using a Monte Carlo approach, in which densities are generated under the null hypothesis of the same number of peaks occurring in random locations. Hence, the densities over the threshold represent clustering of peaks within a brain area over what would be expected by chance if the reported peaks occurred in random locations in the brain.

ALE meta-analysis was carried out using GingerALE 2.0.4 software distributed by the BrainMap project (http://brainmap.org/ale/) [21]. This software models each focus was modelled with a Gaussian function defined by a full-width at half-maximum (FWHM) empirically determined using an algorithm developed by Eickhoff and collegues [67] in order to model the spatial uncertainty of each focus using an estimation on the intersubject and studies variability. Statistical significance was determined through a permutation test of randomly distributed foci. The probability maps were thresholded at p<0.05 and corrected using False Discovery Rates (FDR), in order to reduce false positives [68]. The 3-D coordinates were collected from the studies selected. The meta-analysis was performed in Talairach space. Coordinates reported in Montreal Neurological Institute (MNI) space in the original studies were transformed to the Talairach using the Lancaster transform (icbm2tal software procedure [69]) included in the Convert Foci tool of GingerALE.

## Results

### Resting State Studies on Depression and Anxiety Disorders

**Figure 1:**
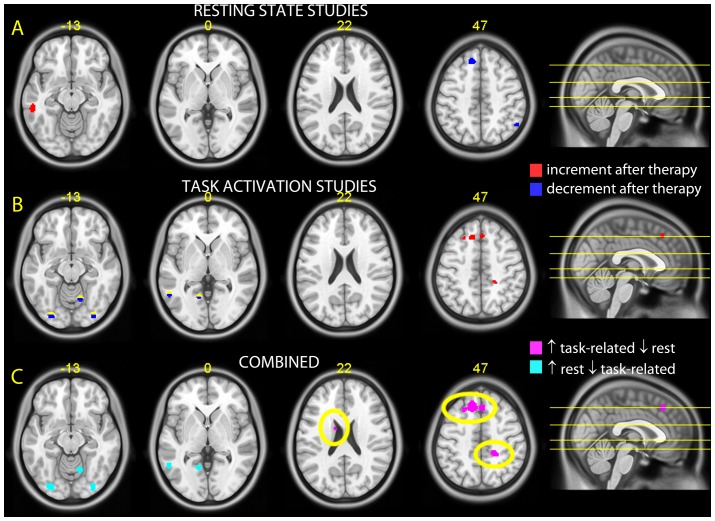
Results. Significant clusters detected in the meta-analyses of rest and task-related studies of the therapy of depression and anxiety, overlaid on a standard template brain. At the top of each transversal brain slice, the number indicates location in Talairach coordinates (in yellow). Slices are also indicated on the right on a sagittal slices. A: resting state studies. In red and blue, increases and decreases of rest activity after therapy. B: task-activation studies. In red and blue, increases of decreases of the contrast of interest task vs. control condition. C: joint analysis. In violet, changes consistent with a reduction of activity during rest and an increase of the difference between task-related and control condition activity. In green, changes consistent with an increase of activity during rest and a decrease of the task-related contrast. Clusters marked with an ellipse highlight clusters of consistent effects across rest and task-activation studies (discussed in text).

The literature contained 6 studies in which the effect of therapy was investigated on changes in resting state. In total, the meta-analysis was conducted on a total of 70 subjects, 50 foci of decreased activation, and 29 foci of increased activation after psychotherapy. Two clusters of decreased activation were found in the fronto-parietal attentional system, one in the left superior and medial frontal gyri, and one in the right inferior parietal lobule (Figure 1A, z = 47). Only one cluster of increased activation was detected by the meta-analysis, located in the inferior and middle temporal lobe (Figure 1A, z = –13). Coordinates of detected clusters are reported in Table 2.

**Table 2:** Significant clusters of increased and decreased activation in emotional-cognitive tasks studies.

Cluster	Cerebral areas	Extrema Talairach Coordinates			Broadmann Areas	Cluster size (mm^3^)	ALE score
		*x*	*y*	*z*			
**Increased activation clusters resting state**							
**1**	Inferior Temporal Gyrus/Middle Temporal Gyrus	–56	–34	–14	20/21	352	0.008
**Decreased activation clusters resting state**							
**1**	Superior Frontal Gyrus/Medial Frontal Gyrus	–12	34	46	8/6	384	0.111
**2**	Inferior Parietal Lobule	48	–56	42	39/40	336	0.009
**Increased activation clusters emotional-cognitive tasks**							
**1**	Superior Frontal Gyrus/Medial Frontal Gyrus/Cingulate Gyrus	–12	28	44	8/32	376	0.013
**Decreased activation clusters emotional-cognitive tasks**							
**1**	Posterior Cingulate/Lingual Gyrus/Cuneus	–10	–58	6	18/19/30	560	0.015
**2**	Inferior Occipital Gyrus/Fusiform Gyrus	–30	–84	–14	18/19	504	0.014
**3**	Inferior Temporal Gyrus/Middle temporal Gyrus	–56	–54	–2	37/21	272	0.013
**4**	Inferior Occipital Gyrus/Fusiform Gyrus	32	–84	–14	18/19	256	0.011
**5**	Cerebellum	14	–62	–14	–	208	0.011
**Decreased activation resting state/Increased activation in emotional-cognitive tasks (new clusters)**							
**1**	Superior Frontal Gyrus/Medial Frontal Gyrus/Cingulate Gyrus	–12	30	46	8/6/32	1760	0.017
**2**	Cingulate Gyrus/Precuneus	18	–36	42	31/5/7	808	0.014
**3**	Superior Frontal Gyrus/Medial Frontal Gyrus	2	30	46	8	360	0.012
**4**	Caudate/Cingulate Gyrus	–14	–2	26	24	328	0.013

Clusters significant at *p*<0.05, false discovery rate (FDR) corrected. Minimum cluster size = 200 mm^3^.

### Studies with Emotional-cognitive Tasks on Depression and Anxiety Disorders

The meta-analyses of emotional-cognitive tasks studies included 5 studies, comprising a total of 65 subjects, and yielding 57 foci of decreased activation and 26 foci of increased activation after psychotherapy. Significant foci of increased and decreased activity are shown in Figure 1B.

As in resting state studies, a cluster of increased task-related activity after psychotherapy was found in the fronto-parietal attentional system (superior and medial frontal gyri, Figure 1B, z = 47).

Several clusters of decreased activation after psychotherapy were also detected. Clusters of decreased activation were located in the posterior cingulated with extensions to the cuneus and lingual gyrus (Figure 1B, z = 0), in the inferior and middle left temporal gyrus (but not in the exact location of the cluster found in resting state studies), bilaterally in visual areas (Figure 1B, z = –13 and 0) and in the cerebellum (Figure 1B, z = 47). Coordinates of detected clusters are reported in Table 2.

### All Studies on Depression and Anxiety Disorders

The analysis in which the resting state studies and the task-related studies were considered together confirmed the results achieved separately for these categories of studies. A notable difference in the pooled analysis consisted of two clusters in the superior and medial frontal gyrus (Table 2). One of them, previously detected in both resting state and task studies considered separately, became more extended because of the contribution of both categories of studies. A second cluster in the medial frontal gyrus emerged for the first time in the conjunct analysis and was located more medially.Finally, new clusters were found in the cingulated gyrus and precuneus (Figure 1C, z = 47) and in the cingulate gyrus with extention to the caudate (Figure 1C, z = 22). After psychotherapy, such areas were less activated in resting state and more activated during task execution.

### Exposure Therapy Studies on Specific Phobia

The meta-analysis on phobic patients treated with exposure therapy included symptoms provocation paradigms. There were 5 studies in this meta-analysis, based on 57 participants and 38 foci. In the meta-analysis of the contrast post- versus pre-therapy, a significant cluster of decreased activation in limbic areas was detected (parahippocampal gyrus and fusiform gyrus (*x* = 30, *y* = –28, *z* = –18; BA 20/35/36; cluster size 360 mm^3^, ALE score 0.008). No significant clusters of increased activation were found in exposure therapy studies.

## Discussion

In the present meta-analysis we evaluated the findings of existing longitudinal studies of brain function changes in psychotherapy in patients with depression or anxiety disorders. Longitudinal neuroimaging studies of both resting state and of emotional-cognitive tasks were included and analysed separately and conjunctly. To the best of our knowledge, this is the first attempt to apply meta-analytic method in summarizing results on neural correlates of psychotherapy change.

According to the dual-process model of affective disorders and their therapy, a change in prefrontal functioning would be expected in comparing patients before and after psychotherapy, reflecting changes in the effectiveness of voluntary emotion regulation processes [10], [32], [70]. The present meta-analysis was designed to test this construct in the effects of psychotherapy assessed with neuroimaging studies of disorders in which the model has received empirical support in the literature. However, no involvement of the DLPFC could be demonstrated, the area most specifically associated with working memory [9], [71] executive attention [72] and with emotion regulation in studies based on the application of voluntary emotional change strategies [10], [73].

There are several, not necessarily mutually exclusive, possible explanations of this null finding. The most obvious explanation would be an insufficient number of studies to demonstrate an effect in the meta-analysis. Even this were true, however, the finding of significant effects elsewhere in the prefrontal lobes or in other parts of the cortex (discussed below) suggests that a more complex model of control may be required to explain the data. A second explanation refers to the relative lack of consistency of the findings of prefrontal involvement in depression and anxiety. For example, previous systematic reviews and meta-analyses of task-related changes in depression have shown the effects in DLPFC to be complex [74], [75]. As these systematic reviews reveal, it seems difficult at present to identify a behaviour-brain circuit that reliably elicits a difference in prefrontal cortex function in affective disorders. A final possible explanation of this null finding may refer to the inadequacy of the voluntary emotion regulation model and its attendant behaviour-brain phenotype to account for psychotherapy effects comprehensively. First, psychotherapists are engaged in different activities, whose effects may not all be adequately represented by the cognitive control/voluntary emotion regulation model. Second, anxiety and depression, even if highly co-morbid and presenting overlapping symptoms and shared neurobiological correlates [19], may also present specific features. For this reason, it may be important for research in this field to develop specific behaviour-brain phenotypes capable to resolve these two disorders, and in future meta-analyses to consider anxiety and depression distinctly. At the present time, not enough studies in each of these groups exist to warrant this separate strategy without incurring in a bias toward null findings.

However, the analysis detected a modulation of prefrontal activity in the DMPFC in both resting state studies and emotional-cognitive tasks studies, even if without a concomitant effect in DLPFC. This finding may be consistent with an emotion dysregulation model of affective disorders remedied by psychotherapy in which improvements in emotional control refer to self-observation and rumination activities, which may be expected to be present both in spontaneous thinking in the absence of an external stimulus to control [76] and during the execution of explicit tasks [77]. Indirect support for this hypothesis is provided by studies that have shown the presence of self-related content to activate this area in the healthy [36], [78], [79] and in association with symptoms of depression [42], [80]. This area is active also in autobiographical memory tasks [81], [82] suggesting its specific involvement when attention is directed toward internal content rather than to external targets. In the present meta-analysis, the effect in this cluster had opposite directions in task-related and resting state studies, with therapy reducing DMPFC activity at rest. This finding would be consistent with reduction of self-referential activity during rest and in the control condition of the task. Another relevant finding in this respect emerges from studies directly comparing voluntary cognitive reappraisal of negative affect and execution of a demanding task in the presence of emotional distractors [11], [12]. While activations in prefrontal areas were largely overlapping in both tasks, an area that was preferentially activated by cognitive reappraisal in both studies was DMPFC. In the interpretive framework given by the findings just reviewed, this preferential medial activation is consistent with the involvement of a process evaluating the relevance of the material for the self, which was not present when emotion regulation followed focus on a demanding cognitive task. Also the cluster detected in the posterior midline cortex in the meta-analysis is consistent with changes in self-referential activity, since the posterior cingulus has been associated with self-referential memory [83], [84].

A second finding of this meta-analysis was the failure to detect significant foci of activity change in limbic areas such as the amygdala in the studies of psychotherapy of depression and anxiety. This finding is disappointing given the evidence showing that this area is usually hyper-activated in depression and anxiety [5], [19]. A possible explanation for this null finding may be that studies that assess the interaction between prefrontal and limbic function by presenting emotional stimuli as distractors in a cognitive task may be less effective in eliciting amygdala activation relative to studies of passive exposure [85], [86]. However, modulation of parahippocampal activity after therapy was found in the exploratory meta-analysis that we conducted on exposure therapies in phobic patients. This finding is at least in part consistent with an emotion regulation model in which therapies act by normalizing reactivity of limbic areas to emotional stimuli, and with a previous meta-analysis of the effects of pharmacological therapy in depression [87]. Furthermore, in this group of studies there were findings of effects in the amygdala, reported as a region of interest without including data for the rest of the brain, that were not included in the meta-analysis for this reason [63]. It is also worth noting that in studies of phobias and exposure therapy there is a close correspondence between the stimulus eliciting symptoms, the target of the psychotherapeutic intervention, and the stimulus that could be used in the experimental setting adopted in the scanner. The high degree of conceptual consistency between symptom forms, model of treatment, and stimuli used in the neuroimaging study may facilitate the identification of a relevant behaviour-brain circuit and the empirical demonstration of therapy change.

Another finding of this meta-analysis was the detection of clusters of significant change in the left inferior and middle temporal cortex increased their activation in the resting state and decreased during task execution. The middle temporal gyrus is most often involved in verbal comprehension [88], social cognition [89] and more generally in semantic processing [90]. With respect of existing models of the neurobiology of depression and its therapy, this finding may be considered unexpected. In a previous meta-analysis of depression and its pharmacological therapy, for example, this area escaped detection [87]. However, a main tenet of psychotherapy strategies, especially for depression, is the reorganization of semantic networks (‘schemas’) [91]. Therefore, this finding also points to the possible lack of comprehensiveness of the voluntary emotion regulation model to account for the effects of psychotherapy, mentioned above. It also suggests questions for future research aimed at identifying possible differences between the effects of pharmacological and psychotherapeutic interventions, as one would not expect reorganization of semantic networks at short term with the former.

The present study cannot be considered as conclusive because of some limitations. A first methodological limitation concerns the amount of data available for analysis. Several studies had small sample sizes, and others did not include suitable control groups in the experiment. Some studies reported only the simple contrast including the variable time (pre- *versus* post-psychotherapy), instead of the interaction between the variable time and the variable group (patients *versus* controls). For this reason it is difficult to conclude that the effects observed are related with psychotherapy treatment, as they may be confounded with the natural course of the condition under therapy. A second limitation ensues from the considerable heterogeneity of techniques and study designs. We consider this limitation unavoidable, as few incentives exist for replicating existing studies exactly. However, considering emotional stimuli presentation in passive viewing or during cognitive tasks execution separately could describe more accurately the mechanisms underlying psychotherapeutic change, and the consideration of disorder-specific stimuli used in the experimental paradigms could be useful in explaining specific psychotherapy effects for specific patients. Yet another limitation concerns studies that had to be excluded from analysis because of reporting only region of interest results. The existence of these reports poses a difficult methodological problem, since including them may bias the analysis toward the regions of interest examined in the original studies, and excluding them may engender the opposite bias. One may hope that the introduction of meta-analytic techniques in this field will have the positive effect of encouraging the adoption of designs and reporting standards that enable the subsequent consolidation of results across studies.

In summary, the present meta-analysis summarized the evidence on the neural correlates of psychotherapy changes. Although some of the results of the present meta-analysis are consistent with some hypothesis proposed in psychotherapy research and neuroscience literature and with studies about neural correlates of psychopathology, the findings considered as a whole suggest a more complex picture than the one posited by the contrast between limbic system and prefrontal areas. The location of identified clusters appears to be most consistent with involvement of self-representational processes in the therapy of depression or anxiety, and with a reduction in reactivity of the medial temporal lobe in exposure therapy of phobias. Other cerebral and mental mechanisms may be involved, such as semantic processes, but more studies are required in order to clarify the influence of these less studied mechanisms on psychotherapy.
